# Dynamic Balance and Muscle Activity in Lifesavers: A Study on Intrinsic and Extrinsic Foot Muscles

**DOI:** 10.7759/cureus.67756

**Published:** 2024-08-25

**Authors:** Kai Suzuki, Tsukasa Kumai, Shota Ichikawa, Toshihiro Maemichi, Takumi Okunuki, Tsunaki Shimpo, Yui Akiyama, Hiroyuki Mitsui, Hisateru Niki

**Affiliations:** 1 Department of Orthopaedic Surgery, St. Marianna University School of Medicine, Kawasaki, JPN; 2 Faculty of Sport Sciences, Waseda University, Tokorozawa, JPN

**Keywords:** dynamic balance, lifesaver, sand training, sand activity, foot muscle

## Abstract

Activities on sandy soil are known to contribute to improved leg strength and balance. Lifesavers (LSs) have shown that sandy soil activity promotes intrinsic foot muscle development and improves balance. LS improves leg strength and balance through activities on sandy soil. However, the effect of foot muscle development on the actual muscle activity of LS remains unclear. We aimed to evaluate the effect of foot muscle development on muscle activity in lifeguards on a sandy beach compared with the corresponding in healthy participants. Fifteen LSs and 15 healthy adults underwent a Y-balance test to assess dynamic balance and surface electromyography to measure muscle activity. The LSs exhibited a significantly higher percentage of maximum voluntary contraction values in the tibialis anterior muscle in all directions than the healthy adults. The LSs showed increased peroneus longus and abductor hallucis muscle activity in the posterolateral and posteromedial directions, suggesting their involvement in contralateral postural control during dynamic balance. These findings suggest that engaging in barefoot activities on sandy soil enhances foot muscle activity and improves dynamic balance in LSs.

## Introduction

Physical activity on sandy soil improves physical functions, such as muscle strength and balance. In the field of sports science, physical activity on sandy soil reportedly improves athlete performance in terms of physical fitness, leg strength, balance function, proprioceptive sensation, and spatial cognitive ability while also reducing the incidence of sports-related injuries [1,2]. Compared with running on land, running on sand increases lower leg muscle activity, particularly during the stride cycle, which requires knee and ankle joint stability [3].

Lifesaving is a sport in which activities are performed on the sand, and participants compete to demonstrate their ability to perform lifesaving activities at sea. Competitive activities include surf events on the coast and beach events, as defined by the International Life Saving Federation. Lifesavers (LSs) work barefoot on beaches and sandy shores. While their physical and physiological functions have been studied [4,5], few studies have evaluated changes in their lower legs and feet in detail [6]. Barefoot activity on sand may affect the foot’s intrinsic muscle cross-sectional area and toe grip strength. However, the effects of foot muscle development on muscular activity and athletic performance in LSs remain unclear. While competition-specific physical activity reportedly improves specific muscle activity patterns and influences balance performance [7], no study has compared the effects of foot muscle development on muscular activity between LSs and healthy participants.

In this study, we aimed to compare dynamic balance and intrinsic and extrinsic foot muscle activities between LSs and healthy participants and to investigate the effects of barefoot sand activity on balance and foot muscle activity.

## Materials and methods

Participants

Lifesaving club members and undergraduate/postgraduate students were recruited via email. The study participants included 15 professional LSs (male, 12; female, 3; mean age, 25.4 ± 2.8 years) and 15 healthy undergraduate/postgraduate students (male, 12; female, 3; mean age, 24.5 ± 5.4 years). Based on a previous study by Ichikawa et al. [6], the study design pre-calculated that 30 participants were required to achieve 80% power, assuming an effect size (d) of 1.1 and an alpha level of 0.05. The healthy participants were assumed to have had no physical activity for the six months preceding their participation in the study. Lifesaving club members who had been active for at least two years were assigned to the LS group. The inclusion criterion comprised age ≥18 years. Individuals who had undergone previous lower-limb surgery, those who experienced lower-limb trauma or disability within six months, and those with a history of diabetes, joint disease, or neurological disease were excluded.

Y-balance test

**Figure 1:**
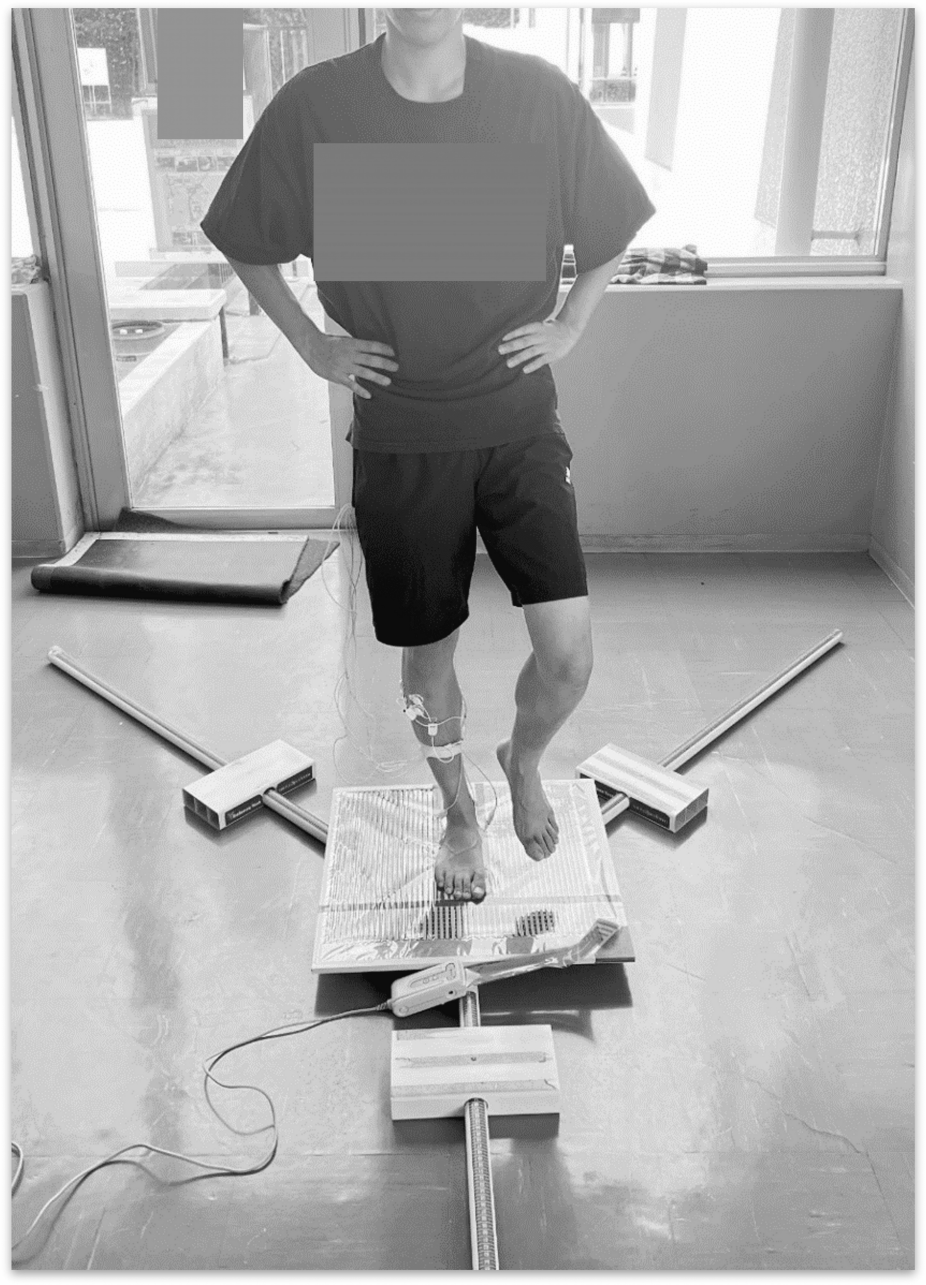
Y-balance test

The maximum reach distance to the anterior (A), posterolateral, and posteromedial (PoM) sides was measured with the right leg supported using a Y-balance test kit (Perform Better Japan, Tokyo, Japan). The measuring limb position was fixed with both hands on the waist. The measurements were taken three times in each direction. If, during the measurement, the extended leg touched the ground, the hand left the waist, or the heel of the weight-bearing foot lifted, the measurement was considered a failure and was repeated (Figure 1).

The maximum reach distance normalized by the leg length was used as a representative value in the analysis. Leg length, defined as the distance from the superior anterior iliac spine to the medial malleolus, was measured using a tape measure [8,9]. The participants maintained the maximum reach position for 1 s, and muscle activity during this period was recorded.

Surface electromyography

Muscle activity during maximum reach was measured and assessed using surface electromyography (biosignalsplux; PLUX Wireless Biosignals S.A., Lisbon, Portugal) with gel-filled self-adhesive disposable Ag/AgCl electrodes (PLUX Wireless Biosignals S.A.). The following muscles were monitored: peroneus longus (PL), tibialis anterior (TA), medial head of gastrocnemius, abductor hallucis (ABH), and abductor digiti minimi. The distance between electrodes was 2 cm in accordance with the method endorsed in the surface electromyography for the non-invasive assessment of muscles (SENIAM) recommendation [10]. Prior to the measurements, two 5-s measurements of maximum voluntary isometric contraction (MVIC) were performed, with the maximum value recorded as the MVIC. In the Y-balance test, the average muscle activity measured during the 1-s maximum reach was normalized in relation to the MVIC, and the average of the three successful trials was used as the representative value.

Statistical analysis

Statistical analysis was performed using IBM SPSS Statistics, version 26.0 (IBM Corp., Armonk, NY) software. Baseline data (age, height, weight, and body mass index), maximum reach distance in the Y-balance test, and muscle activity on electromyography were compared between the two participant groups. The Shapiro-Wilk test was used to test the normality of each variable distribution before between-group comparisons. For normally distributed variables, an unpaired t-test was used; for other variables, the Mann-Whitney U test was used. The significance level was set at p < 0.05. Effect sizes (d) were calculated using G*Power version 3.1.9.7 (Heinrich-Heine-Universität Düsseldorf, Düsseldorf, Germany) software, with values of 0.2-0.49, 0.5-0.79, and ≥0.8 interpreted as small, medium, and large, respectively [11].

## Results

This study included 15 healthy participants without exercise habits and 15 LSs. No significant differences were observed in baseline characteristics between the two groups (Table 1).

**Table 1:** Participant characteristics BMI, body mass index; SD, standard deviation ns, not significant; p > 0.05

Variables	Control group (n = 15; 12 male, 3 female)	Lifesavers (n = 15; 12 male, 3 female)	p-value	Effect size
	Mean ± SD	Mean ± SD		
Age (years)	25.4 ± 2.8	24.5 ± 5.4	ns	0.21
Height (cm)	169.5 ± 6.6	169 ± 7.1	ns	0.07
Weight (kg)	64.7 ± 11.5	66.1 ± 11.4	ns	0.12
BMI (kg/m^2^)	22.4 ± 3.3	23 ± 2.5	ns	0.2

Y-balance test

No significant difference in the maximum reach distance was observed; however, the LS group tended to have higher values in all directions. The effect sizes were moderate for A (d = 0.69) and PoM (d = 0.5) (Table 2).

**Table 2:** Mean reach distance in the Y-balance test A, anterior; PL, posterolateral; PM, posteromedial ns, not significant; p > 0.05

Normalized reach (%)	Control group (mean ± SD)	Lifesavers (mean ± SD)	p-value	Effect size
A	62.1 ± 5.0	65.3 ± 4.3	ns	0.69
PL	110.6 ± 5.7	112.4 ± 6.8	ns	0.28
PM	107.1 ± 6.5	110.4 ± 6.5	ns	0.5

Surface electromyography

During the forward reach measurement, the LS group exhibited significantly higher TA values than those exhibited by the control group (p <0.01), with a tendency toward higher PL values (effect size, d = 0.58). During the posterolateral reach, TA (p < 0.01) values were significantly higher in the LS group, with a tendency toward higher PL values and a greater effect size (d = 0.89). Concerning the posteromedial reach, ABH (p < 0.01) and TA (p = 0.031) values were significantly higher in the LS group, with a tendency toward higher PL values and a medium effect size (d = 0.53) (Table 3).

**Table 3:** Mean EMG activity during the Y-balance test ABH, abductor hallucis; ADD, adductor digiti minimi; A, anterior; GM, medial head of gastrocnemius; PL, peroneus longus; PoL, posterolateral; PoM, posteromedial; SD, standard deviation; TA, tibialis anterior; EMG, electromyography ns, not significant; p > 0.05 *p-value significant at 0.05.

Direction	A				PoL				PoM				
Muscles	Control group (mean ± SD)	Lifesavers (mean ± SD)	p-value	Effect size	Control group (mean ± SD)	Lifesavers (mean ± SD)	p-value	Effect size	Control group (mean ± SD)	Lifesavers (mean ± SD)	p-value	Effect size	
ABH	57.6 ± 40.7	50.2 ± 18.3	ns	0.23	40.6 ± 16.7	45.5 ± 22.9	ns	0.24	17.9 ± 9.7	31.7 ± 15.7	0.004*	1.06	
ADD	40.5 ± 64.9	33.8 ± 49.3	ns	0.12	29.4 ± 15.6	26.1 ± 31.7	ns	0.13	36.5 ± 11.2	29.8 ± 24.4	ns	0.24	
TA	20.7 ± 10.3	40.7 ± 20.6	0.005*	1.23	24.3 ± 13.5	45.3 ± 16.6	0.002*	1.39	35.9 ± 11.2	46.9 ± 12.9	0.031*	0.91	
PL	28.2 ± 13.6	38.4 ± 20.9	ns	0.58	27.4 ± 12.5	41.3 ± 18.4	ns	0.89	27.3 ± 17.4	36.6 ± 17.7	ns	0.53	
GM	8.8 ± 7.6	11.6 ± 9.7	ns	0.32	7.6 ± 5.7	9.9 ± 8.9	ns	0.32	9.1 ± 7.0	9.8 ± 10.1	ns	0.08	

## Discussion

TA muscle activity measured during the star excursion balance test (SEBT) [9,12], which is the original dynamic balance test used in the Y-balance test, is reportedly higher during posterolateral and posteromedial reach in healthy participants [13]. TA muscle contraction produces ankle dorsiflexion, which stabilizes the ankle joint. The ankle dorsiflexion position is a position in which the ankle is stabilized. The ankle dorsiflexion angle in the Y-balance test significantly correlates with the maximum reach during forward reach [14-16]. However, during backward reach, the hip flexion angle has an 88.6-94.5% influence on the maximum reach [14], whereas ankle dorsiflexion has little influence on the maximum reach in the backward position. Karagiannakis et al. [13] reported that the shift in the foot’s center of pressure was greater in the anteroposterior direction than in the lateral direction, irrespective of the direction of reach during SEBT. Shin et al. [17] reported that the TA acted as a brake on the shift in the foot’s center of pressure in the posterolateral direction. Therefore, ankle dorsiflexion may contribute to the control of the center of gravity in the anteroposterior direction during backward reach. In our study, the LSs showed higher TA muscle activity in all directions, suggesting that LSs improve dynamic balance by achieving and maintaining a greater ankle dorsiflexion angle, stabilizing the ankle joint, and countering anteroposterior center-of-gravity displacement. The LSs also tended to have higher PL muscle activity in all directions. Lima et al. [18] reported that five weeks of selective strengthening with electromyostimulation improved the dynamic balance of American football players, with PL and TA muscle activity contributing to extrinsic and intrinsic foot deflections, respectively. The LSs might have maintained more precise plantar leveling and postural stabilization during dynamic balance by simultaneously increasing PL and TA muscle activity. This action helps in controlling the center of gravity in the anteroposterior direction through ankle dorsiflexion.

Few studies have investigated ABH activity during SEBT. In our study, ABH muscle activity tended to be higher during posteromedial reach in the LSs. ABH contraction leads to abduction and flexion at the base of the phalanx and is important for maintaining the medial longitudinal arch of the foot [19]. McKeon et al. [20] showed that the development of intrinsic foot muscles increased the medial longitudinal arch of the foot and improved balance function. Furthermore, LSs have significantly developed intrinsic foot muscles and elevated arches [6,21]. The LSs exhibited greater toe grip strength, with significant correlations between toe grip strength and the maximum distance achieved in the Y-balance test [22]. These muscles are considered to develop in LSs who run barefoot on sandy terrain because they grasp deforming sand with their bare feet and achieve ground contact stability by increasing the density of sand in the plantar region [6]. In this study, the medial longitudinal arch was elevated by the developed ABH in the LSs. The LSs might have maintained a high dynamic balance during posteromedial reach by flexing the hallux and forcefully gripping the ground.

Nagamoto et al. [23] reported that lower-limb mechanical factors and functional impairments, including sensation, balance, and coordination, could affect the maximum reach in the Y-balance test. Olmsted et al. [24] showed that the supporting leg during the SEBT required a sufficient joint range of motion, along with adequate intrinsic sensation and neuromuscular control. Ichikawa et al. [6] reported no differences in terms of foot muscle morphology and muscle cross-sectional areas of the TA and PL between healthy participants and LSs. However, activity on the sandy ground reportedly increases the frequency of co-contraction of periarticular muscles and positively affects neuromuscular factors involved in the efficiency of the muscle contraction-extension cycle [25]. Our study findings indicated that LSs specifically increased the activity of muscles involved in dynamic balance.

Sensory receptors responsible for postural control in the plantar foot region are collectively known as plantar mechanoreceptors [26]. The plantar environment alters the threshold of plantar mechanoreceptors, affecting plantar sensation. Increased plantar sensation improves muscle activity and balance [27,28]. However, in our study, no direct comparison was made between LSs and healthy participants with respect to superficial plantar sensation; thus, further studies are needed.

These findings suggested that barefoot LS on sandy soil may improve dynamic balance by maintaining ankle joint and plantar surface stabilization through efficient foot muscle activity. This new finding suggests that barefoot activity on sandy soil may contribute to the improvement of dynamic balance and efficient muscle strengthening and will contribute to the development of sports and rehabilitation fields.

This study had some limitations. The plantar sensation of the participants was not assessed, and not all extrinsic and intrinsic muscles were measured. Therefore, other factors that could influence dynamic balance were not investigated. A comparison of healthy subjects with a habit of exercise has not been made. Furthermore, lifeguards have not been studied by specialty (sand or swimming) or history of sports activity. Therefore, the effect of the sandy environment on the foot has not been fully investigated. This was an observational study, and it could not be concluded that the sandy environment directly improved dynamic balance. Owing to the small number of study participants, future studies with larger sample sizes are required.

## Conclusions

In this study, we investigated the effects of the sandy environment on the foot using the Y-balance test on LFs. The results showed that LFs controlled their center of gravity in multiple directions, stabilized the leveling of the plantar surface, and maintained high dynamic balance by efficiently using their foot muscles. This study was the first to investigate the dynamic balance ability of LFs by measuring muscle activity in detail. This new finding suggests that sand training may be useful in sports and rehabilitation.
